# Salidroside Improves Bone Histomorphology and Prevents Bone Loss in Ovariectomized Diabetic Rats by Upregulating the OPG/RANKL Ratio

**DOI:** 10.3390/molecules23092398

**Published:** 2018-09-19

**Authors:** Hongxing Zheng, Shanshan Qi, Chen Chen

**Affiliations:** 1 Chinese-German Joint Laboratory for Natural Product Research, College of Biological Science and Engineering, Shaanxi University of Technology, Hanzhong 723000, Shaanxi, China; zhenghongxing@snut.edu.cn; 2 Vitamin D Research Institute, Qinling-Bashan Mountains Bioresources Comprehensive Department C.I.C, Shaanxi University of Technology, Hanzhong 723000, Shaanxi, China

**Keywords:** salidroside, bone, osteoporosis, diabetes, OPG, RANKL, bone turnover markers

## Abstract

Postmenopausal diabetic women have a high risk of fractures. Salidroside has preventive effects on estrogen deficiency-induced osteoporosis and has hypoglycemic effects on diabetes in rats. However, whether salidroside inhibits bone loss in postmenopausal diabetic patients is still unknown. Here, we established a rat model of osteoporosis to investigate the protective effects of salidroside on bone loss induced by ovariectomy combined with diabetes, also investigating the underlying mechanisms. Two-month-old female Sprague-Dawley rats were divided into three equal groups (10 rats in each group): control group (with sham operation, treated with drug vehicle); OVX/T1DM group (ovariectomized diabetic rats); OVX/T1DM-SAL group, comprising ovariectomized diabetic rats treated with salidroside (20 mg/kg body weight) by gavage. The results showed that after 60 consecutive days of treatment, the bone mineral density (BMD) of OVX/T1DM-SAL increased significantly compared with the OVX/T1DM group (*p* < 0.01). The level of serum bone turnover markers, including alkaline phosphatase (ALP), cross linked c-telopeptide of type I collagen (CTX-1), osteocalcin, N-terminal propeptide of type I procollagen (PINP), and tartrate-resistant acid phosphatase 5b (TRACP 5b) were all increased in the OVX/T1DM group compared with the control (*p* < 0.01), and those were decreased by salidroside treatment. Meanwhile, the bone histopathological changes were also attenuated, and the bone marrow adipogenesis was inhibited in salidroside treated rats. Moreover, protein and mRNA ratio of bone osteoprotegerin (OPG)/receptor activator of nuclear factor-κB ligand (RANKL) was upregulated in ovariectomized diabetic rats by salidroside treatment. The results above indicated that the protective effect of salidroside on bone loss induced by ovariectomy and diabetes was mainly due to its ability to suppress bone turnover, inhibit bone marrow adipogenesis, and up-regulate the OPG/RANKL ratio.

## 1. Introduction

Postmenopausal osteoporosis is a metabolic bone disease characterized by loss of bone mass and deterioration of bone microarchitecture. It mainly affects older individuals, especially postmenopausal women, and results in hip and vertebral fractures. Approximately one-half of older women will sustain at least one osteoporotic fracture during their lifetime [1,2]. The decreased level of estrogen post-menopause is the main cause of the bone mass loss [3]. The main clinical symptoms of postmenopausal osteoporosis are reduced bone mineral density (BMD) and increased probability of fracture [4]. Type 1 diabetes mellitus (T1DM) is a chronic disease characterized by the destruction of pancreatic beta cells, inadequate insulin production, and chronic hyperglycemia. T1DM is associated with numerous complications, including disorders of bone metabolism [5], which have significant consequences for patients with T1DM in terms of decreased bone mineral density (BMD) and increased risk of fractures [6,7]. Postmenopausal diabetic women have a high risk of fracture due to estrogen and insulin disorder [8,9]. Estrogen and insulin deficits play an important role in the pathogenesis of osteopenia and osteoporosis. Previous studies have shown additive bone loss in cases of combined estrogen deficiency and hyperglycemia [10,11]. Although estrogen and insulin play important roles in bone turnover, hormone replacement therapy can lead to an increased risk of breast cancer [12,13]. Therefore, other potential treatments for postmenopausal diabetic bone loss are necessary.

**Figure 1:**
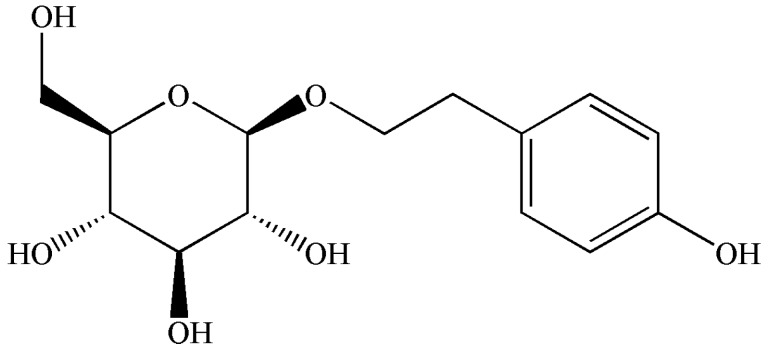
Chemical structure of salidroside.

Salidroside (structure shown in Figure 1) is an active constituent from the root of *Rhodiola rosea* L. that has multiple pharmacological effects, including anti-inflammatory, anti-cancer, and anti-oxidative properties [14,15,16]. Recent studies have shown that salidroside has a protective effect on estrogen deficiency-induced osteoporosis in rats [17]. It also has a hypoglycemic effect on streptozotocin-induced diabetes in rats [18,19]. However, whether it has protective effects on bone loss induced by estrogen deficiency combined with diabetes is still unknown. We hypothesize that salidroside may have a bone-protective effect on bone loss induced by combined estrogen deficiency and diabetes.

The osteoprotegerin (OPG)/receptor activator of nuclear factor-κB ligand (RANKL) axis plays an important role in bone remodeling [20]. OPG and RANKL are produced by osteoblast lineage cells, which are considered to represent a key link between bone formation and resorption during bone remodeling [21]. RANKL stimulates osteoclastic activity and differentiation and inhibits osteoclast apoptosis. OPG inhibits osteoclast formation by binding to RANKL [22]. The bone remodeling process is dependent on the balance of these two proteins, while a low ratio of OPG/RANKL characterizes the increased osteolytic process. The concentration ratio of OPG/RANKL is thus important for preserving bone mass. If this balance becomes skewed, metabolic bone diseases such as osteoporosis can result [23].

Ovariectomized rat is a classical animal model of estrogen deficiency-induced osteoporosis [24]. Streptozotocin is mostly used as a molecule for establishing a T1DM animal model, and both estrogen deficiency and T1DM can induce osteoporosis [25,26]. In this study we established an in vivo ovariectomized (OVX) and streptozotocin-induced osteoporosis model in rats to investigate the protective effects of salidroside on bone loss induced by ovariectomy combined with diabetes, as well as the underlying mechanisms through the OPG/RANKL axis.

## 2. Results

### 2.1. Body Weight

**Figure 2:**
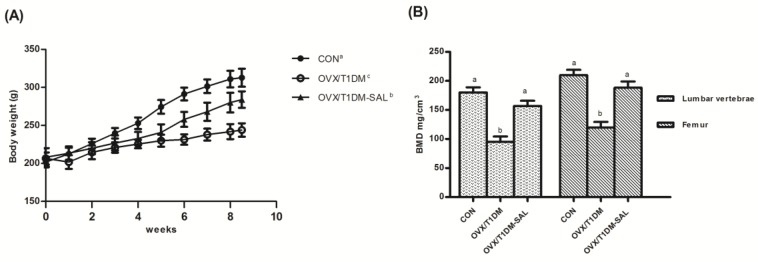
Effects of salidroside (SAL) on (**A**) body weight and (**B**) bone mineral density (BMD) in each experimental group. CON: control group (*n* = 10); OVX/T1DM: group of ovariectomized diabetic rats (*n* = 10); OVX/T1DM-SAL: group of ovariectomized diabetic rats administered with SAL (20 mg/kg/day) (*n* = 10). Values are presented as means ± SD. Different letters indicate statistically significant difference in (**A**) (a, b, c) and (**B**) (a, b; each tissue) (*p* < 0.05, one-way ANOVA followed by Bonferroni post hoc test).

As shown in Figure 2A, there were no difference in the initial body weight of rats in the three experimental groups. Rats with ovariectomy and diabetes (named OVX/T1DM) showed a decreased body weight during the experimental period, but salidroside (SAL) treatment (named OVX/T1DM-SAL) suppressed the decrease in the body weight compared to that observed in vehicle-treated ovariectomized diabetic rats. After 60 days treatment, the final body weight of rats in the OVX/T1DM-SAL group was significantly higher than in the OVX/T1DM group (*p* < 0.05).

### 2.2. Bone Mineral Density Evaluation of Lumbar Vertebrae and Femur

The impacts of salidroside treatment on lumbar vertebrae (L1–L4) and femur bone mineral density (BMD) are shown in Figure 2B. Compared with control rats, BMD in both lumbar vertebrae (L1–L4) and femur were decreased in OVX/T1DM rats (*p* < 0.01), which was significantly recovered by salidroside treatment for 60 days. Additionally, no differences were seen between OVX/T1DM-SAL group and control group, indicating that salidroside had protective effects on BMD.

### 2.3. Serum Glucose, Ca, P, OPG, RANKL, and Bone Turnover Markers

As shown in Table 1, hyperglycemia was observed in the OVX/T1DM group, in contrast to the OVX/T1DM-SAL and control groups. Serum alkaline phosphatase (ALP), c-terminal telopeptide of type 1 collagen (CTX-1), osteocalcin, tartrate-resistant acid phosphatase 5b (TRACP 5b), N-terminal propeptide of type I procollagen (PIPN), and RANKL were significantly higher in the OVX/T1DM group than those in the OVX/T1DM-SAL and control groups (*p* < 0.01). Serum Ca, P, OPG, and OPG/RANKL ratio were significantly lower in the OVX/T1DM group than in the OVX/T1DM-SAL and control (*p* < 0.01).

### 2.4. Morphological and Histomorphometrical Analysis of Femoral Trabecula

**Figure 3:**
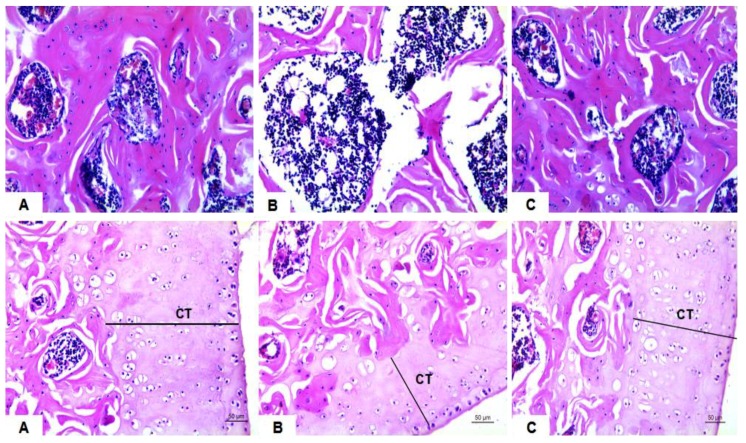
The femoral morphology of rats in each group. (**A**) The femur metaphysis in a rat of control group; (**B**) The femur metaphysis in a rat of OVX/T1DM group; (**C**) The femur metaphysis in a rat of the OVX/T1DM-SAL group. Hematoxylin and eosin staining, magnification: 200×. There was a significant difference in cortical thickness (CT) between control and OVX/T1DM group (*p* < 0.01).

As shown in Figure 3 (A–C, top), ovariectomy and diabetes generated a deteriorated condition of the femoral trabecula according to the histopathological observation. The femoral trabecula was broken and the bone trabecular spacing was increased in the OVX/T1DM group compared with the control. The cortical thickness (CT) was decreased in the OVX/T1DM group (Figure 3B, bottom) compared with the control (Figure 3A, bottom), and it was recovered in the OVX/T1DM-SAL group (Figure 3C, bottom).

**Figure 4:**
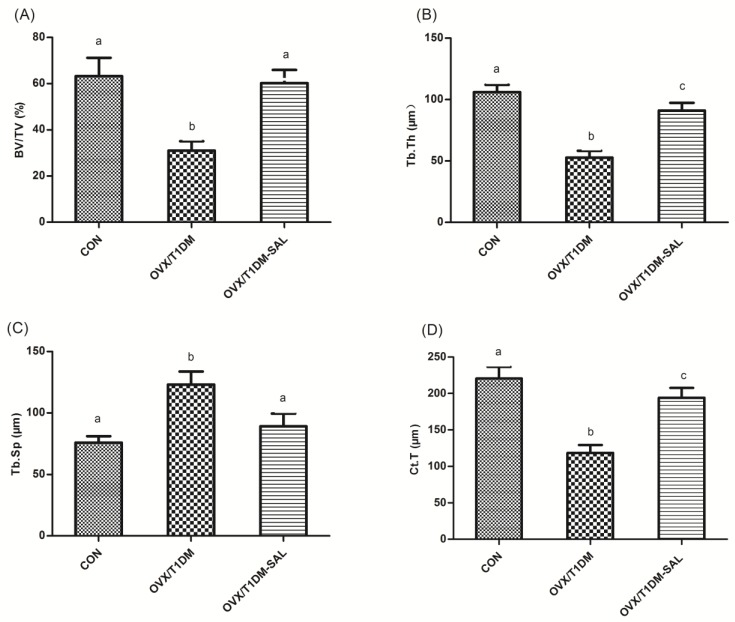
Bone histomorphometric parameters in all experimental groups. (**A**) Bone volume per tissue volume (BV/TV, %); (**B**) Trabecular thickness (Tb.Th, μm); (**C**) Trabecular separation (Tb.Sp, μm); (**D**) Cortical thickness (CT, μm). CON: control group (*n* = 10); OVX/T1DM: group of ovariectomized diabetic rats (*n* = 10); OVX/T1DM-SAL: group of ovariectomized diabetic rats administered salidroside (SAL: 20 mg/kg/day) (*n* = 10). Values are presented as means ± SD. Different letters indicate statistically significant difference (*p* < 0.05, one-way ANOVA followed by Bonferroni post hoc test).

The bone histomorphometric parameters such as bone volume per tissue volume (BV/TV, Figure 4A), trabecular thickness (Tb.Th, Figure 4B), trabecular separation (Tb.Sp, Figure 4C) and cortical thickness (CT, Figure 4D) were all recovered in OVX/T1DM rats with salidroside treatment for 60 days. This result indicates that salidroside could recover the bone structure disorder in rats which was induced by estrogen deficiency combined with diabetes.

### 2.5. Adipocyte Density and Mean Adipocyte Diameter in the Femur Bone Marrow

**Figure 5:**
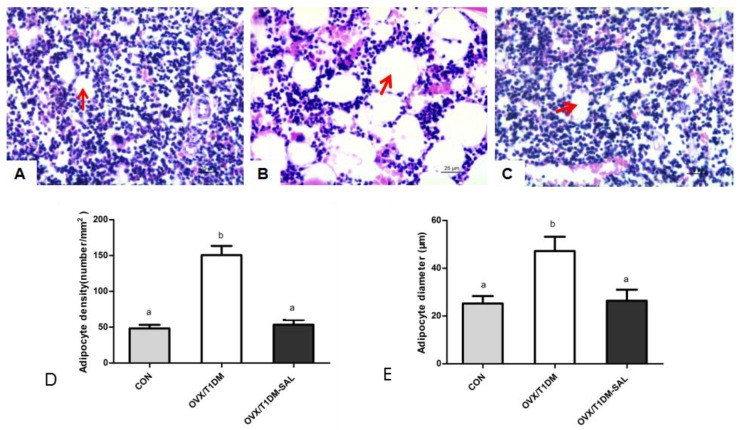
Bone marrow adipocyte parameters in all experimental groups. (**A**) Femur bone marrow in a rat of control group; (**B**) Femur bone marrow in a rat of OVX/T1DM group; (**C**) Femur bone marrow in a rat of OVX/T1DM-SAL group. Hematoxylin and eosin staining, magnification: 400×. (**D**) Adipocyte density of femur bone marrow in each group (*n* = 10 in each group); (**E**) Mean adipocyte diameter of femur bone marrow in each group. Values are presented as means ± SD. Different letters indicate statistically significant difference (*p* < 0.05, one-way ANOVA followed by Bonferroni post hoc test). Red arrows point to adipocytes.

The histological sections showed that the adipocyte number and adipocyte diameter in the femur bone marrow were increased in the OVX/T1DM group (Figure 5B) compared with the control (Figure 5A), and were restored in the OVX/T1DM-SAL group (Figure 5C). As shown in Figure 5D,E, the bone marrow adipocyte density and the mean adipocyte diameter were increased significantly in the OVX/T1DM group compared with the control (*p* < 0.01), and all those parameters were recovered in OVX/T1DM rats with salidroside treatment for 60 days.

### 2.6. The OPG and RANKL mRNA Expression in Bone Tissues, and OPG/RANKL mRNA Ratio in Bone Tissues

**Figure 6:**
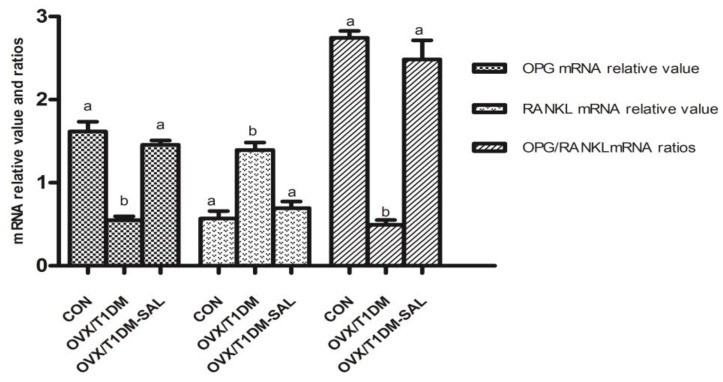
The expression of OPG and RANKL mRNA in bone tissues, and OPG/RANKL mRNA ratio in bone tissues of all experimental groups. CON: control group (*n* = 10); OVX/T1DM: group of ovariectomized diabetic rats (*n* = 10); OVX/T1DM-SAL: group of ovariectomized diabetic rats administered salidroside (SAL: 20 mg/kg/day) (*n* = 10). Values are presented as means ± SD. Different letters (a, b) indicate statistically significant difference in each gene expression group (*p* < 0.05, one-way ANOVA followed by Bonferroni post hoc test).

The mRNA expression analyses are shown in Figure 6. OPG mRNA was decreased, RANKL mRNA was upregulated, and the ratio of OPG/RANKL mRNA was decreased in the OVX/T1DM group compared with the control group (*p* < 0.01), which were totally recovered by salidroside treatment for 60 days (OVX/T1DM-SAL group).

### 2.7. The OPG and RANKL Protein Expression in the Bone Tissues of Rats in Each Group

**Figure 7:**
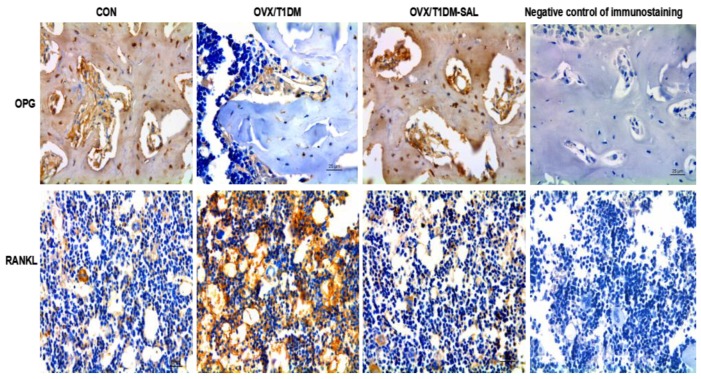
The expression of OPG and RANKL protein in the femoral bone tissues of each group. CON: control group (*n* = 10); OVX/T1DM: group of ovariectomized diabetic rats (*n* = 10); OVX/T1DM-SAL: group of ovariectomized diabetic rats administered with salidroside (SAL: 20 mg/kg/day) (*n* = 10). Negative control of immunostaining: immunostaining was performed using rabbit IgG in place of the primary antibody. In the immunohistochemical staining, the cells with positive expression of OPG and RANKL are shown in brown. Magnification: 200×.

**Figure 8:**
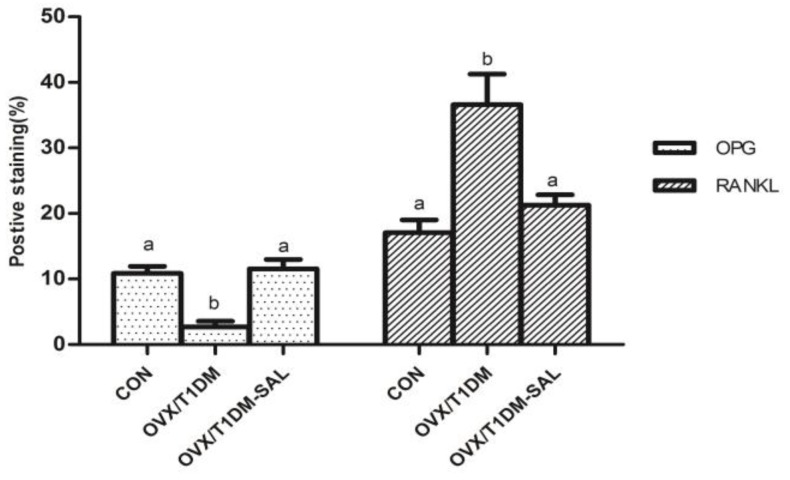
Percentage (%) of the positive staining area of OPG and RANKL in the femoral bone tissues of each group. CON: control group (*n* = 10); OVX/T1DM: group of ovariectomized diabetic rats (*n* = 10); OVX/T1DM-SAL: group of ovariectomized diabetic rats administered with salidroside (SAL: 20 mg/kg/day) (*n* = 10). Values are presented as means ± SD. Different letters (a, b) indicate statistically significant difference in each protein expression group (*p* < 0.05, one-way ANOVA followed by Bonferroni post hoc test).

Immunohistochemical staining showed that the protein expression of OPG was decreased and RANKL expression was increased in the OVX/T1DM group compared with the control. Salidroside supplementation effectively increased the expression of OPG and decreased the expression of RANKL in the bone tissue of OVX/T1DM rats (Figure 7 and Figure 8). As shown in Figure 8, there were no significant differences in percentage (%) of the positive staining area of OPG and RANKL between OVX/T1DM-SAL and control groups (*p* > 0.05). These results indicate that salidroside supplementation could effectively reduce RANKL, and increase both OPG level and OPG/RANKL ratio in the bone tissue of OVX/T1DM rats.

## 3. Discussion

Bone turnover and bone integrity are affected by estrogen deficiency and T1DM [27,28,29]. When both are combined, bone turnover becomes accelerated, and additive bone loss occurs [8,9]. Salidroside (SAL) is a major biologically active compound extracted from the root of *Rhodiola rosea* L. This compound has been used in Traditional Chinese Medicine and has had no reported side effects to-date, including maternal or embryonic toxicity and teratogenic effects [30,31,32]. Studies have shown that salidroside decreases the symptoms of estrogen deficiency-induced osteoporosis [33] and streptozotocin-induced diabetes [19]. However, no evidence has yet suggested that salidroside supplementation is beneficial in cases of bone loss induced by combined estrogen and insulin deficit.

In the present study, we constructed a rat model of osteoporosis induced by both ovariectomy and diabetes. Decreased BMD, bone volume, and cortical thickness, and increased bone turnover biomarkers and trabecular separation were observed in the ovariectomized diabetic rats, indicating the validity of the animal model. After treatment with salidroside for 60 days, the BMD, serum bone turnover markers, and the bone structure of ovariectomized diabetic rats were all recovered, indicating that salidroside was able to ameliorate the severe bone loss induced by estrogen deficiency combined with diabetes.

Bone turnover biomarkers (BTMs) reflect bone formation and resorption, and therefore inform the status of bone remodeling, which is a mechanism underlying osteoporosis [34]. BTMs include both bone formation and resorption markers. Bone formation biomarkers are synthesized by osteoblasts and therefore reflect specific osteoblastic functions, and these include ALP, osteocalcin, and PINP [35]. Increased bone formation markers have been proposed as possible predictors for osteoporosis in postmenopausal women [36,37]. In the present study, the increased bone formation biomarkers (ALP, osteocalcin, and PINP) in the serum of the OVX/T1DM rats may be due to active osteoblasts trying to compensate for the bone loss caused by estrogen and insulin depletion. Bone resorption markers include CTX-I and TRACP 5b [38,39]. Increased CTX-I and TRACP 5b levels have been reported to be inversely correlated with BMD among women [40,41]. In this study, both bone formation (ALP, osteocalcin, PIPN) and bone resorption (CTX-I and TRACP 5b) markers were increased in the ovariectomized diabetic rats, indicating an increased bone turnover, and those BTMs were all restored in the OVX/T1DM-SAL group. Taken together, the data obtained in this study indicate that oral administration of salidroside improved bone quality by suppressing the increase in bone turnover induced by combined estrogen deficiency and diabetes.

Salidroside was recently reported as having the ability to promote the differentiation and mineralization of osteoblasts and decrease the differentiation of osteoclasts [33], thereby exerting osteoprotective effects in ovariectomized (OVX) rats. Consistent with those observations, our study demonstrated that salidroside protected against bone loss and bone deterioration in female rats with estrogen deficiency combined with hyperglycemia, suggesting a potential application of salidroside in managing bone loss in postmenopausal diabetic women. In this study, we found that 60 days of salidroside treatment could effectively increase the BMD, improve the bone tissue structure, and increase the bone volume, and trabecular thickness, and decrease the bone trabecular separation of ovariectomized diabetic rats, supporting our hypothesis that salidroside supplementation is beneficial in combined estrogen deficiency and T1DM induced bone loss. On the other hand, the ovariectomized diabetic rats showed poor bone architecture and quality, suggesting that estrogen deficiency and hyperglycemia trigger a deleterious effect on bone.

Previous research indicated that the number of bone marrow adipocytes is an alternative indicator of the severity of osteoporosis [42,43]. It has been identified as a potential marker or mechanism for diabetes or estrogen deficiency-related skeletal fragility. Greater numbers of marrow adipocytes are associated with lower BMD and increased skeletal fragility [44]. In this study, we first reported increased bone marrow adipocytes and mean adipocyte diameter (μm) in ovariectomized diabetic rats. This increase was prevented by salidroside treatment—after 60 days of salidroside treatment, those parameters were restored. The osteoblasts and adipocytes have common origins in bone marrow [45], and decreased BMD in OVX/T1DM rats may be explained by preferential adipocytic (rather than osteoblastic) differentiation in shared cellular precursors. Salidroside treatment can reverse marrow adipogenesis occurring in OVX/T1DM rats, which may contribute to its capacity to reduce bone loss.

It is well-known that the RANK/RANKL/OPG axis is a critical pathway in maintaining the balance between bone resorption by osteoclasts and bone formation by osteoblasts [46,47]. OPG and RANKL are key factors mediating the differentiation of osteoclasts. OPG is primarily produced by cells of osteoblastic lineage, and has an inhibitory effect on osteoclast formation [48]. Increased RANKL implies an increase of bone turnover related to bone fracture, since RANKL binds with RANK and increases the formation and activation of osteoclast precursors [49]. The OPG/RANKL ratio is important to maintaining an appropriate balance of bone remodeling, which plays a significant role in determining bone mass and skeletal integrity [50]. In the present study, we also assessed the serum OPG and RANKL concentrations, as well as and mRNA and protein expression of OPG and RANKL in bone tissues. We found that after salidroside treatment for 60 days, the serum OPG was increased and serum RANKL was decreased in OVX/T1DM-SAL rats compared with the OVX/T1DM rats. The mRNA and protein expression of OPG and RANKL in bone tissues had a similar pattern of change as serum levels. The serum OPG/RANKL ratio, bone OPG/RANKL mRNA expression ratio, and bone OPG/RANKL protein expression ratio were all increased in the OVX/T1DM-SAL group compared with the OVX/T1DM group, and there were no differences of those indexes between OVX/T1DM-SAL and control groups. These results indicate that salidroside protects against ovariectomy and diabetes induced bone loss by upregulating OPG/RANKL ratio. Our study supports the bone-protective effect of salidroside, and provides preliminary evidence for the potential therapeutic uses of salidroside supplementation in the prevention of bone loss in postmenopausal diabetic women.

## 4. Materials and Methods

### 4.1. Chemicals and Reagents

Salidroside (CAS number: 10338-51-9, purity > 99%, molecular formula: C_14_H_2_O_7_, molecular weight: 300.30) was purchased from Shanghai Ronghe Medicine Science and Technology Development Co., Ltd. (Shanghai, China). Streptozotocin was purchased from Sigma-Aldrich (St. Louis, MO, USA). All other chemicals and reagents were standard commercially available biochemical quality. Water was purified with a Milli-Q purification system and was used to prepare all solutions.

### 4.2. Animals and Treatments

Female Sprague-Dawley rats with age of 8 weeks were purchased from the Laboratory Animal Center of Xi’an Jiaotong University (Xi’an, China). The rats were housed in an air-conditioned room (24 °C) under a 12 h/12 h light/dark cycle. They were fed with standard rodent chow containing 0.9% calcium and 0.7% phosphate and had free access to water. The experiments were conducted after the approval by the Laboratory Animal Administration Committee of the Shaanxi University of Technology, and complied with the Guidelines for Animal Experimentation of the Shaanxi University of technology, the Guidelines on the Care and Use of Laboratory Animals issued by the Chinese Council on Animal Research, and the Guide for the Care and Use of Laboratory Animals published by the National Institutes of Health (NIH publication No. 85-23, revised 2011, New York, NY, USA).

After a 1 week adaptation period, the animals were randomly divided into three groups, and were housed by group (10 rats per group): control, OVX/T1DM, and OVX/T1DM-SAL. The rats in the three groups were anesthetized with pentobarbital sodium anesthesia (30 mg/kg, i.p.). In OVX/T1DM and OVX/T1DM-SAL groups, the bilateral ovaries were excised, blood vessel was ligated, and intraperitoneal wound was sutured. The control rats were subjected to a sham operation procedure wherein only a piece of fat around the ovaries was excised. Fifteen days after surgery, the rats in OVX/T1DM and OVX/T1DM-SAL groups received 60 mg/kg streptozotocin (Sigma-Aldrich Co. LLC, Salvador, Brazil) as reported previously [51]. Equal volumes of vehicle (0.1 mol/L sodium citrate buffer) were injected into the control rats intraperitoneally. After 1 week, blood glucose was measured to confirm the diabetic state, blood samples were obtained from the tail vein, and the glucose concentration was measured using an ACCU-CHEK advantage glucometer (Roche Diagnostics, Indianapolis, IN, USA). Rats with blood glucose concentrations ≥250 mg/dL were considered diabetic. Clinical diabetic symptoms of polyphagia, polydipsia, polyuria, and weight loss were also monitored. In OVX/T1DM-SAL group, rats were given intragastric SAL (20 mg/kg body weight) for 60 days. SAL was dissolved in deionized water, SAL supplementation was started after streptozotocin (STZ) injection. No rats regurgitated it, and no adverse effects were observed during the SAL supplementation period. The supplemental dose of SAL was chosen based on the study of Zhang et al. [33]. In the OVX/T1DM group, rats were giving equal amount of deionized water instead of SAL.

### 4.3. Assay of Ca, P, OPG, RANKL, and Bone Turnover Markers in Serum

At the end of the experiment, rats were weighed and anaesthetized with sodium pentobarbital. Blood was collected by cardiac puncture, and was centrifuged at 2000 rpm at 4 °C for 15 min. Serum samples were stored at −80 °C for biochemical evaluation. Serum calcium and phosphorus concentrations of each sample were analyzed by atomic absorption spectrometer (AAS-900T, Perkin Elmer, Waltham, MA, USA). Serum alkaline phosphatase (ALP), c-terminal telopeptide of type 1 collagen (CTX-1), osteocalcin, procollagen type I N-terminal propeptide (PINP), tartrate-resistant acid phosphatase 5b (TRACP 5b), osteoprotegerin (OPG), and receptor activator of nuclear factor-κB ligand (RANKL) concentrations were determined using an enzyme-linked immunosorbent assay (ELISA) kit (Beijing North Biotechnology Co., Beijing, China).

### 4.4. Bone Mineral Density (BMD) Detection

The lumbar vertebrae and left femur of rats were dissected, and the soft tissue were removed after blood collection. BMD at left femur and lumbar vertebrae (L1–L4) were detected by dual-energy X-ray absorptiometry (DEXA) using a PIXImus 2 (Lunar, Madison, WI, USA) adapted for measuring small animals.

### 4.5. Bone Histopathology and Histomorphometric Analysis

The histomorphometric analysis was performed following a methodology previously described from our laboratory [52,53]. The right femurs of rats were removed and fixed in sodium phosphate (PBS) buffered 4% paraformaldehyde pH 7.4 for 24 h, then the bone tissues were decalcified using 10% EDTA solution for 28 days. Longitudinal 5-μm sections were cut and stained with hematoxylin and eosin for histological examination. The trabecular thickness (Tb.Th, μm), trabecular separation (Tb.Sp, μm), bone volume per tissue volume (BV/TV, %), and cortical thickness (CT, μm) were measured by a computer program (Image-Pro Plus Version 5.0, Media Cybernetics, Inc., Rockville, MD, USA).

### 4.6. Bone Marrow Adipocyte Parameters Analysis

The hematoxylin and eosin-stained slides of femur bone were examined under the microscope. Adipocyte number (number/mm^2^) and mean adipocyte diameter (μm) in the femur bone marrow of each group were measured by a computer program (Image-Pro Plus Version 5.0) according to the method of Kurabayashi et al. [54,55].

### 4.7. Immunohistochemistry for OPG and RANKL Detection

Femur slides wit 5 μm thickness were bathed in citric acid buffer solution (10 mM, pH 6.0) in a 14 kW microwave for 15 min for antigen retrieval, blocked using 3% BSA. Primary antibodies for OPG and RANKL (Invitrogen, Carlsbad, CA, USA) with an optimal dilution (1:200) were allowed to incubate at 37 °C in a moist chamber for 1 h. The negative control for immunostaining was performed using rabbit IgG in place of the primary antibody. The slides were incubated with horseradish peroxidase enzyme labeled secondary antibodies at room temperature for 1.5 h, then incubated with DAB substrate solution, and counterstained with hematoxylin. Images were captured using an inverted microscope (DMI 4000B, Leica, Wetzlar, Germany) The percentage (%) of the positive staining area over the whole field of view was quantified with a computer program (Image-Pro Plus Version 5.0) under ×200.

### 4.8. RT-PCR Analysis of OPG and RANKL mRNA Expression in Bone Tissues

The bone tissue was taken out from the refrigerator at −80 °C, immediately placed into a mortar with liquid nitrogen, then ground. The bone tissue was ground to powder and then transferred to a pre-cooled centrifuge tube. Total RNA of bone tissues was extracted using Trizol reagent (Invitrogen, Carlsbad, CA, USA) according to the manufacturer’s protocol. The cDNA was synthesized using the PrimeScript™ RT Master Mix (TaKaRa, Dalian, China). The expression levels of OPG and RANKL in bone tissues were quantified using real-time PCR, and the primers used are listed below. OPG (635 bp): Forward 5′-TGA GTG TGA GGA AGG GCG TTAC-3′, Reverse 5′-TTC CTC GTT CTC TCA ATC TC-3′ (60 °C). RANKL (750 bp): Forward 5′-ATC AGA AGA CAG CAC TCA CT-3′, Reverse 5′-ATC TAG GAC ATC CAT GCT AAT GTT C-3′ (55 °C). β-actin (240 bp): Forward 5′-GGG CAC AGT GTG GGT GAC-3′, Reverse 5′-CTG GCA CCA CAC CTT CTA C-3′ (55 °C). GAPDH was used as a housekeeping gene for normalization. The reaction conditions were as follows: 95 °C for 10 min; 40 cycles of 95 °C for 10 s, the annealing temperature indicated by primers for 20 s, and 72 °C for 30 s; and 4 °C for 5 min. All experiments were repeated at least three times. The relative change in gene expression was analyzed by 2^−ΔΔ*C*T^ method.

### 4.9. Statistical Analysis

The sample size was confirmed based on a power analysis with a power of 80%. Data were expressed as mean ± standard deviations. Normal distribution was confirmed by Kolmogorov–Smirnov tests. The groups were compared using one-way analysis of variance (ANOVA) followed by Bonferroni post hoc test (using the SPSS computer software Version 18.0, SPSS Inc., Chicago, IL, USA). Differences were considered significant at *p* < 0.05.

## 5. Conclusions

This is the first report demonstrating the preventive effect of salidroside on severe bone loss in ovariectomized diabetic rats. This study demonstrated that salidroside can suppress increased bone turnover, inhibit bone marrow adipogenesis, and upregulate bone OPG/RANKL expression ratio, thus increasing the BMD of the ovariectomized diabetic rats. Salidroside could effectively improve the bone quality in ovariectomized diabetic rats. In the future, we will test whether salidroside has an effect on pancreas and liver, and whether the bone protective effect of salidroside depends on energy metabolism.

## Figures and Tables

**Table 1:** Serum glucose and serum bone turnover markers in each experimental group.

Parameter	Control	OVX/T1DM	OVX/T1DM-SAL
Glucose (mg/dL)	90.46 ± 8.14 ^a^	444.92 ± 29.16 ^b^	102.39 ± 11.82 ^a^
ALP (U/dL)	102.23 ± 9.71 ^a^	189.56 ± 17.33 ^b^	121.67 ± 12.13 ^a^
Ca (mg/dL)	9.41 ± 0.98 ^a^	4.78 ± 0.57 ^b^	8.87 ± 0.91 ^a^
P (mg/dL)	6.54 ± 0.79 ^a^	3.76 ± 0.43 ^b^	5.01 ± 0.91 ^c^
CTX-1 (ng/mL)	21.43 ± 3.78 ^a^	104.32 ± 13.45 ^b^	30.78 ± 4.91 ^a^
Osteocalcin (ng/mL)	19.56 ± 3.59 ^a^	37.42 ± 4.89 ^b^	27.31 ± 3.18 ^c^
TRACP 5b (U/L)	1.74 ± 0.20 ^a^	3.02 ± 0.67 ^b^	1.97 ± 0.43 ^a^
PINP (μg/L)	40.35 ± 7.56 ^a^	51.21 ± 8.54 ^b^	42.67 ± 6.89 ^a^
OPG (ng/mL)	6.78 ± 1.90 ^a^	2.45 ± 0.56 ^b^	5.74 ± 0.69 ^a^
RANKL (ng/mL)	1.89 ± 0.56 ^a^	4.17 ± 0.98 ^b^	1.99 ± 0.51 ^a^
OPG/RANKL ratio	4.21 ± 0.54 ^a^	0.62 ± 0.11 ^b^	3.78 ± 0.37 ^a^

ALP: alkaline phosphatase; Ca: calcium; P: phosphorus; CTX-1: c-terminal telopeptide of type 1 collagen; TRACP 5b: tartrate-resistant acid phosphatase 5b; PINP: N-terminal propeptide of type I procollagen; OPG: osteoprotegerin; RANKL: receptor activator of nuclear factor-κB ligand; CON: control group (*n* = 10); OVX/T1DM: group of ovariectomized diabetic rats (*n* = 10). Values are presented as mean ± standard deviation. Different letters within rows indicate statistically significant difference (*p* < 0.05, one-way ANOVA followed by Bonferroni post hoc test).
